# Exploring the Dimensionality of the Social Skills Improvement System Using Exploratory Graph Analysis and Bifactor-(*S* − 1) Modeling

**DOI:** 10.1177/1073191120971351

**Published:** 2020-11-16

**Authors:** Margarita Panayiotou, Joãο Santos, Louise Black, Neil Humphrey

**Affiliations:** 1 University of Manchester, Manchester, UK

**Keywords:** social skills improvement system, SSIS, exploratory graph analysis, network analysis, bifactor modeling

## Abstract

Since its development over a decade ago, the Social Skills Improvement System (SSIS) has been one of the most widely used measures of social skills in children. However, evidence of its structural validity has been scant. The current study examined the original seven-factor and more recent five-factor structure (SSIS-SEL) of the self-report SSIS in a sample of English elementary school students (*N* = 3,331) aged 8 to 10 years (*M* = 8.66, *SD* = 0.59). A problematic fit was found for both structures with poor discriminant validity. Using exploratory graph analysis and bifactor-(*S* − 1) modeling, we found support for a four-factor structure, the variation of which was captured by a general factor defined by “empathy and prosocial skills.” Future researchers, particularly those interested in using specific domains of the SSIS, are urged to assess its structure in their studies, if their findings are to be theoretically meaningful.

Social skills are behaviors needed for the successful completion of social tasks, such as making friends, playing a game, and initiating conversation (Gresham et al., 2010). Children and young people with social skills deficits are at increased risk of experiencing far-reaching difficulties in and out of school. Considerable evidence has shown that such deficits are a key characteristic of developmental and academic difficulties (Blair et al., 2015; Elias & Haynes, 2008; Malecki & Elliot, 2002; Montroy et al., 2014; Rabiner et al., 2015; Racz et al., 2017; Spence, 2003; Verboom et al., 2014; Wentzel, 1991), with their impact extending far into adulthood (Jones et al., 2015).

Social skills can be developed and improved (Gresham & Elliott, 2008) and explicit instruction of social skills is a core component of programs that focuses on the treatment of emotional, behavioral, and developmental disorders (Spence, 2003). Indeed, meta-analyses of multicomponent programs focused on enhancing—among others—the social skills of children and adolescents report reduced behavioral and emotional problems, and more favorable social behaviors, school bonding, and academic attainment (Durlak et al., 2010; Durlak et al., 2011; Taylor et al., 2017). Identifying children with or at risk of social skills deficits using appropriate instruments has thus become a key focus of intervention efforts, given that the assessment of such skills plays a crucial role in the design of appropriate interventions (Elliott et al., 2008).

## The Social Skills Improvement System

The Social Skills Improvement System (SSIS; Gresham & Elliott, 2008) is one such instrument. The SSIS represents a comprehensive and improved revision of the widely used Social Skills Rating System (SSRS; for a detailed comparison see Gresham et al., 2011), and it is the first measure to directly link assessment to social skills interventions (Frey et al., 2011). The SSIS provides the opportunity to assess social skills, problematic behaviors and academic competence through ratings from teachers, carers, and students (Gresham & Elliott, 2008). The current study focuses exclusively on the social skills strand of the student self-report SSIS, which purports to assess skills of communication (e.g., *saying please*), cooperation (e.g., *paying attention when others talk*), empathy (e.g., *feeling bad when others are sad*), assertion (e.g., *asking for information*), responsibility (e.g., *careful with other people’s belongings*), engagement (e.g., *getting along with others*), and self-control (e.g., *staying calm when teased*).

The SSIS was shown to be psychometrically superior to the SSRS (Gresham et al., 2011). However, unlike the SSRS, the reduction of items and their assignment into each of the seven domains was not based on statistical methods such as exploratory factor analysis (EFA). Instead, its structure was driven by theory and empirical evidence (Frey et al., 2011). While EFA can be an important and informative step in scale development, it is not uncommon for confirmatory factor analysis (CFA) to be performed instead of EFA when prior theory exists about a measure’s structure (Henson & Roberts, 2016). However, when a seven-factor CFA was conducted in the original validation study (Gresham & Elliott, 2008), it resulted in a poor model fit (CFI = mid80s) with modification indices suggesting multiple cross-loadings. Despite this, the structure was not revised or investigated further. The authors report that the “purpose of this analysis was not to test a factor model, but rather to identify possible beneficial changes in subscale composition” (Gresham & Elliott, 2008, p. 51). While such revisions were considered, the authors decided against them as this would have “reduced the number of items loading on each factor, which in turn would have reduced the reliability of the factor” (Gresham & Elliott, 2008, p. 51). While a few studies have explored the measure’s reliability and validity (Cheung et al., 2017; Frey et al., 2014; Gamst-Klaussen et al., 2016; Gresham et al., 2010; Gresham et al., 2011; Sherbow et al., 2015), its structural validity remains largely underexplored, with the limited available evidence pointing to a weak structure.

Using a polytomous IRT model Anthony et al. (2016) found 19 items of the teacher-report SSIS to perform poorly. Similarly, while fit was acceptable according to some indices, the comparative fit and Tucker–Lewis indices were below recommended cutoffs in a Chinese sample. The seven subscales were also shown to be redundant, as they failed to contribute sufficient explanatory variance beyond the total score (Wu et al., 2019). In a recent effort to advance social and emotional learning (SEL) measurement using the original standardization data (*N* = 224), Gresham and Elliott (2018) reconfigured the 46-item SSIS into a five-factor structure representing the five competencies of CASEL’s (Collaborative for Academic, Social, and Emotional Learning [CASEL], 2008) SEL framework: self-awareness, self-management, social awareness, relationship, and responsible decision making. Despite the promising applications of such a measure, Gresham et al. (2020) found inconsistent model fit (root mean square error of approximation [RMSEA] = .06, comparative fit index [CFI] = .83) and poor discriminant validity for the self-report version, with 8 out of 10-factor correlations exceeding .85. Similar findings were found by Panayiotou, Humphrey, and Wigelsworth (2019) in a sample of 7- to 10-year-old English students, with an inadmissible structure due to poor discriminant validity (factor correlations >1).

### The Current Study

Since its development a decade ago, the SSIS has continued to receive increased attention and use in the field of SEL, with over 400 citations; however, the validity of its structure, especially for the self-report version, remains a neglected area of enquiry. This is a significant oversight, given the increased use of self-report assessment in research and clinical practice, in line with policy that focuses on the voice of the child (Deighton et al., 2014; Sturgess et al., 2002). Extending the work by Panayiotou et al. (2019), and as encouraged by the authors of the measure (Frey et al., 2011), the current study aims to examine the structure of the student-report SSIS using secondary analysis of a major data set of English students. The fit of the original seven-factor measure and the newly proposed five-factor SSIS-SEL are assessed, and the structure of the measure is further explored with the use of a new and powerful exploratory analytic technique (exploratory graph analysis [EGA]; Golino & Epskamp, 2017).

## Method

### Design

The current study is based on a secondary analysis of baseline data drawn from a major randomized trial of a school-based preventive intervention.

### Participants

Given that the self-report SSIS was originally validated in a sample of children aged 8 years and older and its readability grade was shown to be 1.8 (Gresham & Elliott, 2008), we excluded any children that were in Grade 1 during the first year of data collection. Of the original sample (*N* = 5,218), the current study included 3,331 students (male; *n* = 1,720, 51.6%) aged 8 to 10 years (*M* = 8.66, *SD* = 0.59). Their characteristics mirrored those of students in state-funded English elementary schools, albeit with larger percentages of students eligible for free school meals (28.6%), speaking English as additional language (21%) and with special educational needs (20.7%; Department for Education, 2012, 2013). Similar to the national picture (Department for Education, 2012), 68.8% of the sample were Caucasian (*n* = 2,292), 11.3% Asian (*n* = 376), 7% Black (*n* = 233), 5.6% mixed ethnicity (*n* = 187), 2.4% other/unclassified ethnicity (*n* = 80), and 0.6% (*n* = 20) Chinese. Ethnic background data were not available for the remaining 143 (4.3%) students.

### Measures

The self-report SSIS for ages 8 to 12 years was used in the current study (Gresham & Elliott, 2008). Students are asked to indicate how true a statement is for them using a 4-point scale (“never,” “seldom,” “often,” “almost always”) and the 46 items are summed to represent a total social skills score or seven individual domains of social skills (communication, cooperation, empathy, assertion, responsibility, engagement, and self-control). This version was originally shown to have acceptable internal consistency and test-rest reliability for the overall scale (α = .94, *r =* .80) and for the seven subscales (α range .72-.80; *r* range = .58-.79; Gresham & Elliott, 2008).

### Procedure

Following approval from the authors’ host institution ethics committee, written consent was sought from schools’ head teachers. Opt-out consent was sought from parents, and opt-in assent from participating students. Data collection took place between May and July (summer term) of 2012. Data were collected electronically via a secure online survey site. School staff supported any students with literacy difficulties to enable them to complete the measure.

### Data Analysis

#### Existing Structures

The original SSIS structure and SSIS-SEL were tested in M*plus* 8.3 using the weighted least squares with mean and variance adjusted (WLSMV) estimator, as this is optimal with ordinal data using large number of latent factors and large sample sizes (Muthén et al., 2015). Model fit was assessed using multiple indices as generally recommended; specifically the CFI, Tucker–Lewis index (TLI), RMSEA; including 90% confidence intervals [CIs]), and standardized root squared mean residual (SRMR). TLI and CFI values above .95, RMSEA values below .06, and SRMR values below .08 were considered to indicate good model fit (Hu & Bentler, 1999). Modification indices and the residual correlation matrix were also assessed for areas of misfit. Given that students were nested within schools (*n* = 45; intracluster correlation coefficients = .004-.063), goodness-fit-statistics and standard errors of the parameter estimates were adjusted to account for the dependency in the data (using Type = complex).

#### New Structure

EGA (Golino et al., 2020; Golino & Epskamp, 2017) is part of network psychometrics, a rapidly developing field that estimates the relationships between observed variables rather than treating them as functions of latent variables (Epskamp et al., 2016). In these models, nodes (circles) represent the observed variables, and edges (lines) represent their level of connection, as partial correlations, after conditioning on all other variables in the model (Epskamp & Fried, 2018). EGA first applies a Gaussian Graphical Model (Lauritzen, 1996) to estimate the network by modelling the inverse of the variance–covariance matrix (Epskamp et al., 2017). Then, using penalized maximum likelihood estimation (graphical least absolute shrinkage and selection operator [glasso]), the model structure and parameters of a sparse inverse variance–covariance matrix are obtained (Golino & Epskamp, 2017). glasso uses a tuning parameter to minimize the extended Bayesian information criterion, thus estimating the most optimal model fit (Epskamp & Fried, 2018). glasso is useful in avoiding overfitting, by shrinking small partial correlation coefficients, and can therefore also increase the interpretability of network structures (Epskamp et al., 2016). Finally, the walktrap algorithm (Pons & Latapy, 2006) is used to find how many dense subgraphs (clusters) exist in the data. These clusters are considered to be mathematically equivalent to latent variables (Golino & Epskamp, 2017). As with other traditional exploratory factor analytic methods, EGA is data driven and does not rely on the researcher’s a priori assumptions or beliefs, making it an ideal technique for testing or reevaluating the structure of a measure (Christensen et al., 2019). Traditional factor analytic methods follow a two-step approach, where the number of dimensions is estimated first, for instance through parallel analysis, followed by EFA with the number of dimensions found in Step 1. EGA on the other hand, offers an advantage over traditional methods, as it follows a single-step approach, thus reducing the number of researcher degrees of freedom and the potential for bias and error (Golino et al., 2020). It was also shown to outperform parallel analysis and minimum average partial procedure, especially in models with highly correlated factors, such as the SSIS (Golino et al., 2020; Golino & Epskamp, 2017).

To examine the structure of the SSIS, the sample was split into two random halves, one for EGA (*n* = 1,666) and one for CFA (*n* = 1,665). The EGA was performed using the R package *EGAnet* (version 0.9.3; Golino & Christensen, 2019), which makes use of the *qgraph* package to estimate the polychoric correlations and glasso. The network model and CFA models were visualized using the R packages *qgraph* (version 1.6.3; Epskamp et al., 2012) and *semPlot* (version, 1.1.2; Epskamp et al., 2019), respectively.

CFA, which was used to evaluate the EGA structure found in previous steps, was estimated using M*plus* 8.3. Data were analyzed using WLSMV with pairwise presence, and accounting for the school clustering. Model fit was determined based on the criteria aforementioned. The code for all analyses is provided in the supplementary material.

## Results

### Seven-Factor SSIS Structure

The correlated seven-factor structure was shown to have acceptable model fit in the current sample (*N* = 3,331), χ^2^ (968) = 2479.394, *p* < .001; RMSEA = .023 (90% CI [.021, .024]), CFI = .928, TLI = .924; SRMR = .043. While the CFI and TLI values were somewhat below the acceptable thresholds, it is known that these indices can be worsened by having a large number of indicators (Kenny & McCoach, 2003). Acceptable factor loadings (λ range = .42-.76) were observed across most SSIS items. However, a correlation of 1 between responsibility and cooperation resulted in a nonpositive psi matrix. Furthermore, as shown in Table 1, seven pairs of factor correlations indicated poor discriminant validity with *r* > .80 (Brown, 2015). A nonpositive psi matrix was also observed for the second-order structure due to a negative but small residual variance for the responsibility factor (ξ = −.006) and a factor correlation >1 between responsibility and the second-order factor. While this was resolved when the residual variance was fixed to zero, as with the lower order model, eight pairs of factor correlations were shown to be substantially large (see Table 1). The fit of this model, χ^2^(983) = 2848.616, *p* < .001; RMSEA = .025 (90% CI [.024, .026]), CFI = .912, TLI = .907, SRMR = .048, and the clear evidence for poor discriminant validity, pointed to a possible misspecified solution.

**Table 1.** Factor Correlations for the Correlated (Left Diagonal) and Higher Order (Right Diagonal) Seven-Factor Models.

	1	2	3	4	5	6	7
1. Communication	—	.848	.793	.950	.823	.770	.786
2. Cooperation	.901	—	.744	.892	.772	.722	.737
3. Assertion	.745	.639	—	.834	.722	.676	.690
4. Responsibility	.945	1.002	.791	—	.866	.810	.826
5. Empathy	.867	.755	.757	.824	—	.701	716
6. Engagement	.732	.627	.835	.736	.735	—	.669
7. Self-control	.751	.719	.729	.854	.681	.715	—

*Note.* All correlations were significant at the .001 level.

### SSIS-SEL Structure

As a next step, the structure of the newly proposed SSIS-SEL was considered in the same sample. Results pointed to two issues. First, as with the original study (Gresham et al., 2020), this model was shown to have inconsistent fit, with respect to CFI and RMSEA, χ^2^(979) = 3355.421, *p* < .001; RMSEA = .028 (90% CI [.027, .029]), *p* > .05; CFI = .888, TLI = .881, SRMR = 053. Strictly following the minimum acceptable cutoffs, it appeared to fit the data well in terms of RMSEA, but poorly when CFI was considered. Given the limitations of fit indices (McNeish et al., 2018), the aim is not to blindly disregard the model, but try to explain why such discrepancy might have occurred. When investigating the residual correlation matrix in the current data, only a relatively small percentage of correlations (9.7%) were equal or greater than .10 (Kline, 2016). This, along with the SRMR index, indicates that the level of misfit is low. In this case, some suggest that inconsistent model fit may be due to high measurement accuracy (i.e., low unique variances; Browne et al., 2002). However, communalities ranged between .14 and .58 within our data, with the majority (63%) being below .40 (Costello & Osborne, 2005). Accordingly, the unique variance was high for most items (ε = .42-.86) indicating that while the level of misfit is low, many of the items are not meaningfully related to their respective factors. In such instances, further exploration of the factor structure is warranted (Costello & Osborne, 2005).

The second issue was that the psi matrix was again nonpositive definite, caused by the substantially high correlations between the five factors, ranging between .74 (social awareness × self-management) and 1.01 (self-awareness × responsible decision making), with 9 out of 10 correlations exceeding .80. Therefore, the degree to which the five SEL factors represent distinct constructs is questionable. While it would be possible to collapse the highly correlated factors in an effort to improve fit, the model misspecifications noted above could arise from an improper number of factors (Brown, 2015). While CFA relies on a strong theoretical background, as was the case for the development of the SSIS, factor misspecifications “should be unlikely when the proper groundwork for CFA has been conducted” (Brown, 2015, p. 141). Given the lack of exploratory techniques during the initial stages of the SSIS development, we thus sought to further explore the structure of the measure within our data.

#### New Structure

**Figure 1.**
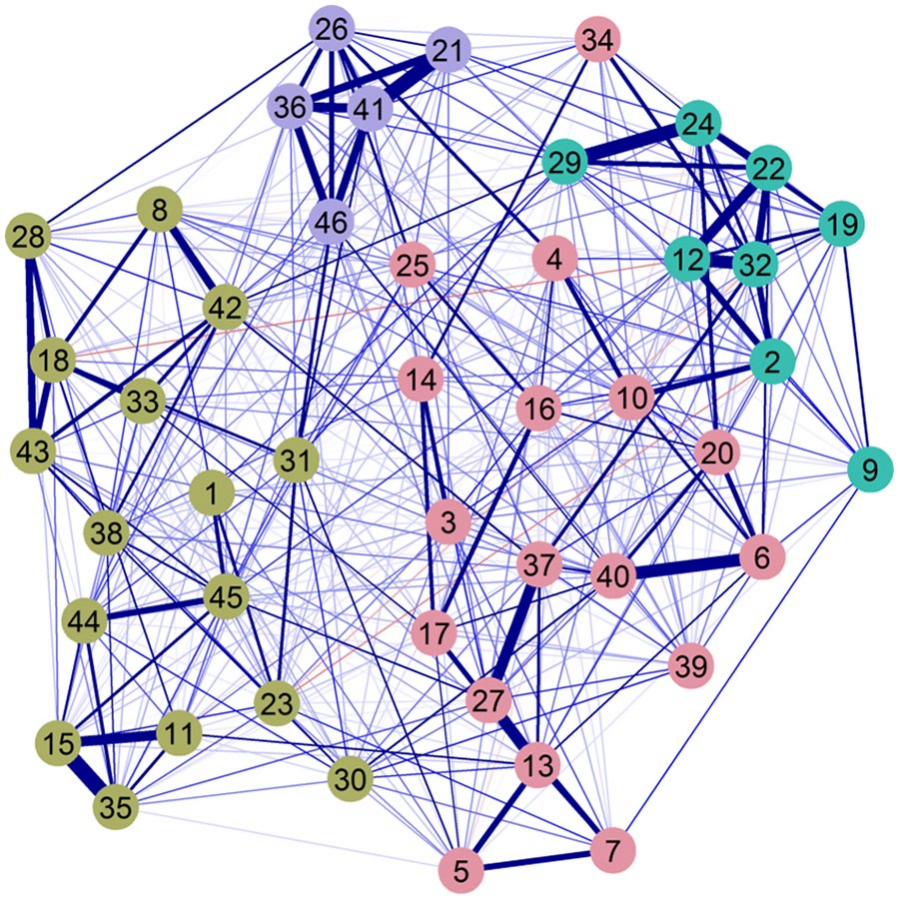
Exploratory graph analysis of the 46 SSIS items, with each color representing one cluster (latent variable). *Note*. Nodes (circles) represent observed variables, and edges (lines) represent partial correlations. The magnitude of the partial correlation is represented by the thickness of the edges. SSIS = Social Skills Improvement System; blue edges = positive correlations; red edges = negative correlations.

**Figure 2.**
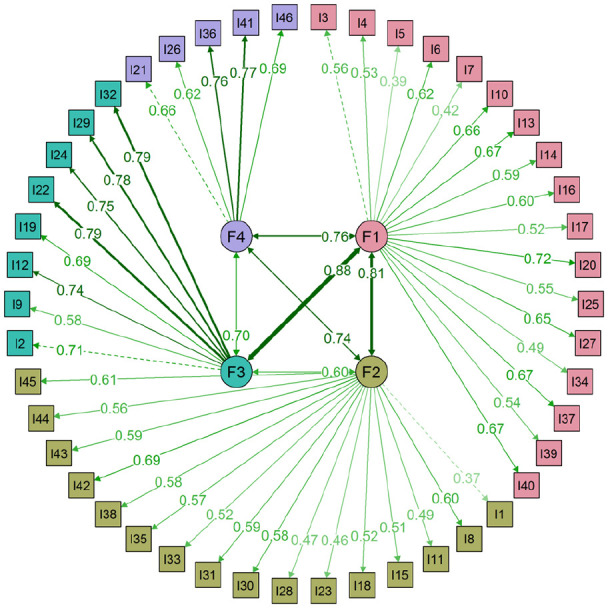
Confirmatory factor analysis of the four-factor EGA structure. *Note*. EGA = exploratory graph analysis; F1 = empathy and prosocial skills; F2 = engagement and relationship skills; F3 = cooperation; F4 = self-control.

EGA of the 46 items (*n* = 1,666) resulted in a dense network with four clusters of partial correlations (see Figure 1). CFA of this four-factor EGA structure in the second random half (*n* = 1,665) showed acceptable model fit, notably better than the original seven-factor and five-factor SEL structure, χ^2^(983) = 1601.943, *p* < .001, RMSEA = .020, (90% CI [.019, .022]), CFI = .955, TLI = .953, SRMR = .043. The majority of residual correlations approached zero and only 38 (3.7%) were ≥ .10, indicating low misfit. It is worth noting, however, that as seen in Figure 2, 28 of the items had communalities below .40 (with λ < .63) and for seven of these this was below .25 (with λ < .50; Costello & Osborne, 2005). Finally, two-factor correlations were above .80, still indicating issues with discriminant validity. Specifically, F2 was shown to correlate most strongly with all other factors (ψ = .76-.88). The four factors were considered to represent empathy and prosocial skills ([F1] 17 items, ω = .85), engagement and relationship skills ([F2] 16 items, ω = .84), cooperation ([F3] 8 items; ω = .84), and self-control ([F4] 5 items; ω = .79). A comparison between this four-factor and existing seven- and five-factor structures can be found in the appendix.

##### Post hoc bifactor models

**Figure 3.**
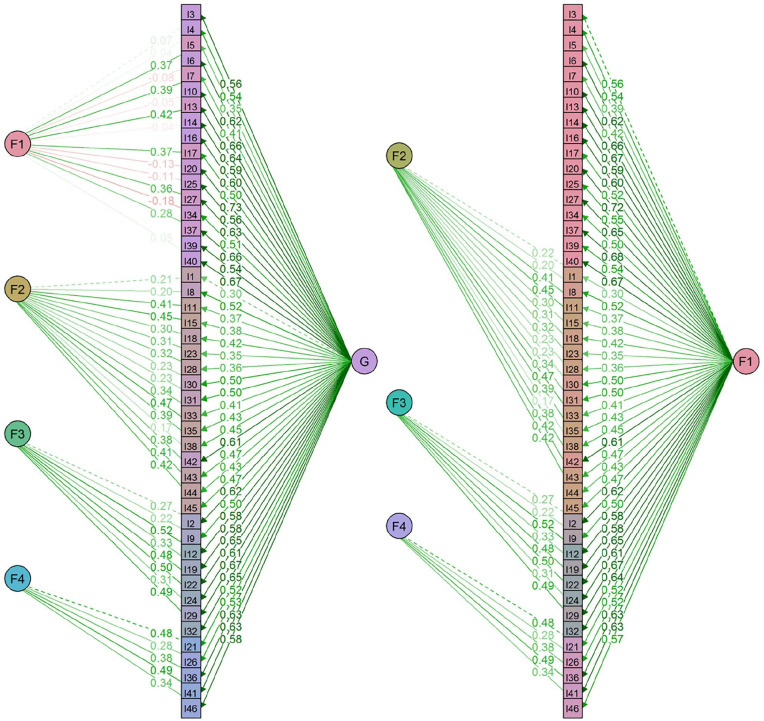
Classical bifactor and bifactor-(S − 1) models of the SSIS. *Note*. SSIS = Social Skills Improvement System.

Given the persistent high factor correlations in the four-factor structure, a bifactor model was examined (Reise et al., 2010). In this model, the covariance between the items can be accounted for by a common general factor (g) and specific domain factors, alongside measurement error (Reise, 2012). This allowed further exploration of the dimensionality of the SSIS and estimation of the extent to which any differences in social skills are determined by a common factor or by specific components (Bornovalova et al., 2020; Eid et al., 2018). While this model was shown to have a good fit, χ^2^(943) = 1477.129, *p* < .001, RMSEA = .019, (90% CI [.017, .021]), CFI = .961, TLI = .957, SRMR = .040, this was not used to guide model selection, given the overfitting issues associated with bifactor modeling (Bonifay et al., 2017; Greene et al., 2019). Most items had moderate to strong factor loadings onto g (λ = .30, .73, *p* < .001, *Μ*_λ_ = .53) and all items, with the exception of i5, i11, i15, and i35, loaded more strongly onto the general than their respective factors (see Figure 3). This resulted in 4 weak specific factors that each mostly reflected a few indicators (Bornovalova et al., 2020). Once the general factor was accounted for, F1 (empathy and prosocial skills) resulted in an uninterpretable pattern of irregular loadings with a near-zero nonstatistically significant variance (ψ = .004, *p* > .05). This is a common problem occurring in bifactor modeling, whereby a factor vanishes and the general factor becomes specific to the set of items for which the factor vanished (Eid et al., 2017; Eid et al., 2018; Geiser et al., 2015). In such cases, applying a bifactor-(*S* − 1) model is recommended, as these models avoid estimation problems and provide a clear interpretation of the g factor (Eid et al., 2017). Bifactor-(*S* − 1) is a reconfiguration of the classical bifactor model, where one specific factor is omitted. In this model, the general factor is defined by the omitted (reference) factor and the specific factors capture variation in the items that are not accounted for by the general factor (Eid et al., 2018).

A bifactor-(*S* − 1) with empathy and prosocial skills (F1) as the general reference factor resulted in acceptable fit χ^2^(960) = 1583.320, *p* < .001, RMSEA = .021, (90% CI [.019, .022]), CFI = .955, TLI = .951, SRMR = .043 (see Figure 3). The variances of the three factors were positive and statistically significant, though very small variances were observed for F2 (ψ = .05) and F3 (ψ = .08), due to the small factor loadings of the reference indicators. It is important to note that while for simplicity the first indicator is typically used for the identification of the latent factor, in theory, the variance of F2 and F3 could take any value between .02 to .22, and .05 to .27, respectively, depending on the choice of reference indicator. Out of the 29 items in the three specific factors, 26 (90%) loaded more strongly on the general empathy and prosocial skills factor (GEP). Most items had factor loadings above the minimum threshold of .30-.40 (Brown, 2015) on the specific factors, though only 11 were shown to exceed .40 (see Table 2). Thus, it is noteworthy, that while all factor loadings were, as expected, positive and statistically significant, small factor loadings were observed for some items on all three specific factors.

**Table 2.** SSIS Bifactor-(S − 1) Factor Loadings and Indices.

	λ_G_	λ_S_	Con	Spe	ECV	ω	ω_h_	H	FD
*Reference g: Empathy and prosocial* (ψ = .31)					.77	.96	.90	.95	.97
i3: Forgive	.56								
i4: Careful with other’s belongings	.54								
i5: Stand up for others	.39								
i6: Say please	.62								
i7: Feel bad for others	.42								
i10: Take turns	.66								
i13: Make others feel better	.67								
i14: Do my part	.59								
i16: Look at people	.60								
i17: Help friends	.52								
i20: Polite	.72								
i25: Self-awareness	.55								
i27: Think of other’s feelings	.65								
i34: Do homework	.50								
i37: Nice to others	.68								
i39: Keep promises	.54								
i40: Say thank you	.67								
*F2: Engagement and relationship skills* (ψ = .05)					.63	.87	.32	.69	.83
i1: Ask for things	.30	.22	.92	.08					
i8: Get along	.52	.20	.95	.05					
i11: Show how I feel	.37	.**41**	.56	.44					
i15: Say I have a problem	.38	.**45**	.54	.46					
i18: Make friends	.42	.30	.83	.17					
i23: Invite others	.36	**.31**	.77	.23					
i28: Talk to new friends	.36	**.32**	.75	.25					
i30: Smile or wave	.50	.23	.93	.07					
i31: End disagreement	.50	.23	.92	.08					
i33: Play games	.41	**.34**	.77	.23					
i35: Say when not treated well	.43	.**47**	.53	.47					
i38: Ask to join	.45	**.39**	.70	.30					
i42: Work well with others	.62	.17	.97	.03					
i43: Make new friends	.47	**.38**	.72	.28					
i44: Tell people-mistakes	.44	.**42**	.64	.36					
i45: Ask for help	.47	.**42**	.69	.31					
*F3: Cooperation* (ψ = .08)									
i2: Listen to others	.62	.27	.91	.09	.69	.90	.26	.63	.83
i9: Ignore naughty	.50	.22	.95	.05					
i12: Do what teacher asks	.58	.**52**	.62	.38					
i19: Do work	.58	**.33**	.86	.14					
i22: Follow school rules	.65	.**48**	.69	.31					
i24: Behaved	.61	.**50**	.66	.34					
i29: Do right thing	.67	**.31**	.90	.10					
i32: Listen to teacher	.65	.**49**	.68	.32					
*F4: Self-control* (ψ = .23)					.67	.83	.27	.50	.77
i21: Stay calm-teased	.52	.**48**	.50	.50					
i26: Stay calm-mistakes	.53	.28	.87	.13					
i36: Stay calm-problems	.63	**.38**	.73	.27					
i41: Stay calm-bothered	.63	.**49**	.56	.44					
i46: Stay calm-disagreement	.58	**.34**	.79	.21					

*Note.* The item numbering corresponds to the SSIS Rating Scales, and descriptions reflect the content of the items but are abbreviated to avoid copyright violations. In bold are items with factor loadings >.30. Underlined are items that load more strongly onto their own domain. SSIS = Social Skills Improvement System; λ_G_ and λ_S_ = factor loadings on the general and specific factors, respectively; Con = consistency; Spe = specificity; ECV = explained common variance; ω_h_ = hierarchical omega reliability, H = construct reliability; FD = factor determinacy. All factor loadings were statistically significant (*p* < .001).

Key bifactor indices were also computed, using the R package *BifactorIndicesCalculator* (version 0.2.0; Dueber, 2020). Given that these were originally developed for the classical bifactor model, only the following that could be extended to bifactor-(*S* − 1) were considered (Rodriguez et al., 2016). The explained common variance represents the proportion of common variance that is due to g (Rodriguez et al., 2016). For the specific factors, this is interpreted as the proportion of common variance of the items in *a specific factor* that is due to g. Omega (ω) internal consistency estimates the *combined* reliability of g and specific factors, while *hierarchical* omega (ω_h_) represents the reliability of a factor after controlling for the variance due to g (Rodriguez et al., 2016). Construct reliability (H) provides the variance of the factor that is accounted for by the items and can also be interpreted as a reliability coefficient (Hancock & Mueller, 2001). Factor determinacy (FD) represents the correlation between factor scores and the factors, with values closer to 1 indicating better determinacy (Grice, 2001). Finally, the consistency and specificity of the bifactor-(*S* − 1) items were considered. Consistency provides the proportion of the true score variance of a nonreference item that is determined by the reference factor, while the sensitivity of an item estimates its true score variance that is not determined by the reference factor (1-consistency; Eid et al., 2017).

Omega and H values >.70 were considered acceptable (Hancock & Mueller, 2001; Rodriguez et al., 2016), and only factors with FD > .90 were considered reliable for using factor scores as their proxy (Gorsuch, 1983). Results are summarized in Table 2. The explained common variance and omega reliability coefficients indicated that the majority of the variance in the SSIS was explained by GEP. Overall, GEP met the recommended thresholds for omega reliability, FD and H in our sample, but this was not the case for the three specific factors, which were shown to explain very little variance. Notably, while the omega reliability coefficient was shown to be high for the specific factors (ω = .83-.90), this was substantially lower once the variance associated with GEP was partitioned out (ω_h_ = .26- .32). Results were further supported by the consistency and specificity of the nonreference items, which showed that 50% to 97% of their variance was accounted for by GEP.

## Discussion

The aim of the current study was to assess, for the first time, the dimensionality of the self-report SSIS in a sample of English elementary school students. Both the current and original studies, as well as that for the revised SSIS-SEL, found a poor fitting model with low CFI values (Gresham & Elliott, 2008; Gresham et al., 2020; Panayiotou et al., 2019). While, in the current study, this might have been the result of low communalities, our evaluation of the SSIS structure did not rely exclusively on fit index cutoffs, as good practice suggests (McNeish et al., 2018). In fact, the most important finding of the original studies, as well as the current one, is the measure’s poor discriminant validity. In many instances, the correlations between factors neared or exceeded 1, indicating that they generally fail to assess distinct constructs (Brown & Moore, 2012). This was in line with findings by Wu et al. (2019), who found that the seven factors were redundant and did not explain much variance beyond the total score. This issue is of relevance particularly when researchers are interested in specific sets of skills, and are using the SSIS factors as independent stand-alone scales. In fact, the findings reported here challenge the idea that these factors can be used to measure seven distinct constructs.

From a statistical perspective, excessively high factor correlations might be the result of overfactoring (Brown & Moore, 2012). This was supported in our sample of English students, where robust analyses pointed to a four-factor solution. These findings are, to a great extent, unsurprising, given that exploratory analysis of the SSIS and SSIS-SEL was not conducted during development. While it is true that EGA is a data-driven approach and, as the authors note, “CFA is most appropriate for theory testing rather than for theory generation as is done in exploratory factor analysis” (Gresham et al., 2020, p. 197), their stance can become highly problematic under certain conditions. When said CFA techniques result in poor model fit and statistically indistinguishable factors, this might be the result of improper number of factors due to the lack of robust exploratory groundwork (Brown, 2015). At this stage, model revision based on robust exploratory methods might be more appropriate.

Additionally, premature use of CFA might be inappropriate with measures that are based on inconsistent conceptualization of the construct under study. As many researchers have noted, the area of social and emotional development suffers from “jingle and jangle fallacies,” where different definitions are used to assess the same skills, resulting in great measurement challenges (Abrahams et al., 2019; Jones et al., 2016). Social functioning, for instance has been considered an umbrella term for “social competence” and “social skills,” but other times these have been used interchangeably (Cordier et al., 2015). Future researchers are therefore urged to take such challenges into consideration and test the structure of the measure in their own sample prior to testing structural differences. In the current sample, most items of the original “engagement” and “assertion” domains clustered together and were considered to represent “engagement and relationship skills.” Its factor loadings were, however, varied (λ = .37-.70; h^2^ = .22-.49), suggesting that not all items are well explained by this factor, potentially also leading to issues with sum scores (McNeish & Wolf, 2020). The remaining three factors in our four-factor structure were considered to represent “self-control” (e.g., *stay calm when teased*), “cooperation” (e.g., *well behaved*), and “empathy and prosocial skills” (e.g., *make others feel better*). While the four-factor structure was shown to have a better model fit than the original one, issues with discriminant validity remained. Specifically, the empathy and prosocial skills factor was shown to correlate very highly with all domains (ρ = .76-.88), further questioning the dimensionality of this construct.

Though the aim of the current study was not to revise the self-report SSIS, EGA provides a unique account of the relationships between the 46 items and allows for a deeper understanding of possible problematic areas in the structure. While the dense network of the current study (Figure 1) resulted in four clusters, a few of the strong correlations between items of different domains (e.g., i2 × i10), and items that deviate from their cluster (e.g., i30, i34), could explain the poor discriminant validity observed in the current structure (the full partial correlation matrix is provided in Table S1, Supplementary material, available online). EGA can thus provide a detailed representation of how the items from each cluster relate to one another, but also how these clusters are placed with each other in multidimensional space (Christensen et al., 2019).

Concerns about the measure’s unclear dimensionality were confirmed by the post hoc bifactor findings. While bifactor models have received increased attention within psychology in the past decade, their application within social skills has been scant. Our analyses were therefore necessarily exploratory. Consistent with the greater literature, the classical bifactor structure for the SSIS resulted in inadmissible results (Eid et al., 2017). Specifically, in the presence of g, the factor loadings of F1 were uninterpretable and irregular, causing the specific factor to vanish. Within stochastic measurement theory such findings are expected when classical bifactor models are applied to structures with noninterchangeable (fixed) domains, such as those studied here (Eid et al., 2017). Bifactor-(*S* − 1), on the other hand, has proved promising, and been applied to many psychological constructs, without the problems inherent to classical bifactor models (e.g., Black et al., 2019; Burns et al., 2020; Eid, 2020; Heinrich et al., 2018). While it is advised that the choice of reference factor in bifactor-(*S* − 1) is based on theory and/or ease of interpretation (Burns et al., 2020; Eid, 2020), our decision was empirically driven: F1 was shown to drive the high factor correlations in the correlated-factor model and it vanished in the classical bifactor model. Given that the meaning of g varies depending on the choice of reference factor (Burke & Johnston, 2020; Burns et al., 2020), results should be interpreted with caution, as generalizability cannot be assumed.

The empirical collapse of F1 in this study fits with the work by Zachrisson et al. (2018) who treated “prosocial behavior” as their reference factor in the Lamer Social Competence in Preschool scale, under the assumption that this is the most aligned domain with overall social competence. Within our sample, social skills were captured by GEP and the three specific factors were considered to represent systematic variation among items that cannot be captured by the reference factor (Eid et al., 2018). It is therefore important to remember that neither the reference nor the specific factors represent overall social skills, as this study initially set out to explore. Rather, the specific factors indicate that a child exhibits more or less self-control, cooperation, and engagement and relationship skills, than one would expect given his or her levels of GEP. This aligns with theoretical and empirical evidence that suggests empathy is a key driver of prosocial behavior (Decety et al., 2016), and that prosocial behavior is directly related to self-regulatory behaviors, social interactions and general social competence (e.g., Andrade et al., 2014; Dewall et al., 2008; Spinard & Eisenberg, 2009). Indeed, in the current sample 50% to 97% of the variance in the items was captured by GEP, while the specific factors explained very little variance and were shown to be psychometrically unfit. Additionally, it is important to note that given the small factor loadings, the meaning captured by the specific factors becomes fundamentally different (Bornovalova et al., 2020). F3 for example, no longer captures general cooperation skills, but cooperation within school and classroom, if one were to use a threshold of >.40 for meaningful factor loadings.

Overall, our findings and those reported elsewhere (Gresham & Elliott, 2008; Gresham et al., 2020; Panayiotou et al., 2019) suggest that currently the SSIS is unable to meaningfully capture distinct domains of social skills (or social–emotional competence in the case of SSIS-SEL). This does not necessarily mean, however, that the poor discriminant validity of the measure is the result of a common general factor. Positive manifolds between symptoms and behaviors mathematically fit bifactor models but might represent processes other than a common cause (van Bork et al., 2017). For instance, the positive manifold observed in the current study might be the result of measurement problems (van der Maas et al., 2006). Based on sampling theory (Bartholomew et al., 2009), it is possible that it may be very difficult to obtain independent measures of different groups of social skills, as these rely on the same underlying behaviors (van Bork et al., 2017; van der Maas et al., 2006). This has been true for all the SSIS structures examined in the current sample, as it was proven difficult to obtain structurally independent domains of social skills. This overlap could be because different sets of skills are tapping onto the same underlying processes, in this case empathy and prosocial behavior. Given that the SSIS was developed through focus groups with professionals and teachers (Gresham & Elliott, 2008), one must also consider whether this overlap is caused by a gap between young children and scale developers in their ability to differentiate between such highly overlapped items. Thus, before concluding that empathy and prosocial skills can sufficiently explain the covariance between the SSIS items, more work is needed to understand how to disentangle these overlapping skills both conceptually and statistically. Indeed, some of the difficulties reported in the current study are consistent with general problems in the conceptualization of social–emotional learning. This suffers from inconsistent and variable scope and psychometric properties (Humphrey et al., 2011; Wigelsworth et al., 2010). However, as this is the first study to explore the structure of the self-report SSIS, more work is needed to consolidate the results reported herein. Until said work is carried out, for researchers using the self-report SSIS that are interested in specific domains of social skills, our findings suggest that a four-factor structure (see Figure 2), might be more appropriate than the original seven-factor structure. However, given that in our study two of the domains (F1 × F3) indicated substantial overlap, we urge researchers to explore the factor intercorrelations in their own data before accurate conclusions can be drawn.

### Strengths and Limitations

Despite being a very widely used measure, the psychometric properties of the SSIS have been a neglected area of inquiry. The current study is the first to explore the structure of the self-report SSIS since its development a decade ago. While the aim of this study was not to revise the SSIS, the application of robust analyses such as the EGA and bifactor-(*S* − 1) allowed us not only to explore the psychometric performance of the SSIS but also possibly shed light on our understanding of social skills more generally. Results from the current study provide robust evidence that currently the SSIS is not fit for the assessment of distinct domains of social skills. Although it is possible that results are culturally specific, the poor psychometric evidence for the seven-factor structure in the current study matched that of the original U.S. standardization sample. Our study is the first to validate the SSIS in a sample of English children, making a significant contribution on the replicability of its structure, further suggesting that even in English-speaking countries, the cultural transferability of the SSIS cannot be assumed (Humphrey et al., 2011). Given, however, that the current study relied exclusively on self-report data, findings cannot be generalized to other SSIS informant types. Future work is thus urgently needed to replicate the results of the current study to the parent and teacher forms and in different cultures and samples. Additionally, given recent findings that glasso might be less powerful in reducing false-positive rates (Williams & Rast, 2020), future work should consider adapting EGA with other nonregularized methods. Finally, any conclusions drawn are limited to the current sample, given that the classical bifactor model is sensitive to sampling (Bornovalova et al., 2020) and the meaning of bifactor-(*S* − 1) is conditional on the chosen reference factor. Nevertheless, findings from attention-deficit/hyperactivity disorder and oppositional defiant disorder research suggest that consistent results are a possibility within bifactor-(*S* − 1) (Eid, 2020). Therefore, more work is needed to identify whether there is a consistently outstanding general domain of social skills that could be used within clinical assessment.

## Conclusion

The findings of the current study add new and robust evidence about the psychometric quality and, specifically, the structural validity of the SSIS measure. In the first validation study of the SSIS in an English sample of elementary school students, the current study rigorously demonstrated that the proposed seven- and five-factor structures of the SSIS are problematic and the 46 items are better represented by a four-factor structure, that are captured through a general reference factor of empathy and prosocial skills. Future researchers, especially those interested in using distinct domains of the SSIS, should consider using the four-factor structure found here, but are also urged to confirm this structure in their own sample, if their findings are to be theoretically meaningful. A better structure on the SSIS could improve the assessment and monitoring of children’s social skills and deficits, and “ultimately contribute to their well-being, resiliency, and achievement of adaptive outcomes” (Abrahams et al., 2019, p. 468).

## Supplemental Material

Supplementary – Supplemental material for Exploring the Dimensionality of the Social Skills Improvement System Using Exploratory Graph Analysis and Bifactor-(S − 1) Modeling Click here for additional data file. Supplemental material, Supplementary for Exploring the Dimensionality of the Social Skills Improvement System Using Exploratory Graph Analysis and Bifactor-(S − 1) Modeling by Margarita Panayiotou, Joãο Santos, Louise Black and Neil Humphrey in Assessment

## Appendix

**Table A1.** The Existing and Newly Proposed Structures of the SSIS.

Items	SSIS factors		
	EGA four-factor	Seven-factor	Five-factor SEL
i1	Engagement and relationship skills	Assertion	Relationship skills
i2	Cooperation	Cooperation	Self-awareness
i3	Empathy and prosocial	Empathy	Relationship skills
i4	Empathy and prosocial	Responsibility	Responsible decision making
i5	Empathy and prosocial	Assertion	Social awareness
i6	Empathy and prosocial	Communication	Self-awareness
i7	Empathy and prosocial	Empathy	Social awareness
i8	Engagement and relationship skills	Engagement	Relationship skills
i9	Cooperation	Cooperation	Self-management
i10	Empathy and prosocial	Communication	Relationship skills
i11	Engagement and relationship skills	Assertion	Social awareness
i12	Cooperation	Cooperation	Relationship skills
i13	Empathy and prosocial	Empathy	Social awareness
i14	Empathy and prosocial	Responsibility	Self-awareness
i15	Engagement and relationship skills	Assertion	Self-awareness
i16	Empathy and prosocial	Communication	Relationship skills
i17	Empathy and prosocial	Empathy	Social awareness
i18	Engagement and relationship skills	Engagement	Relationship skills
i19	Cooperation	Cooperation	Self-management
i20	Empathy and prosocial	Communication	Self-awareness
i21	Self-control	Self-control	Self-management
i22	Cooperation	Cooperation	Responsible decision making
i23	Engagement and relationship skills	Engagement	Relationship skills
i24	Cooperation	Responsibility	Self-awareness
i25	Empathy and prosocial	Assertion	Self-awareness
i26	Self-control	Self-control	Self-management
i27	Empathy and prosocial	Empathy	Social awareness
i28	Engagement and relationship skills	Engagement	Relationship skills
i29	Cooperation	Responsibility	Responsible decision making
i30	Engagement and relationship skills	Communication	Relationship skills
i31	Engagement and relationship skills	Self-control	Self-management
i32	Cooperation	Cooperation	Self-management
i33	Engagement and relationship skills	Engagement	Relationship skills
i34	Empathy and prosocial	Responsibility	Responsible decision making
i35	Engagement and relationship skills	Assertion	Self-awareness
i36	Self-control	Self-control	Self-management
i37	Empathy and prosocial	Empathy	Social awareness
i38	Engagement and relationship skills	Engagement	Relationship skills
i39	Empathy and prosocial	Responsibility	Responsible decision making
i40	Empathy and prosocial	Communication	Relationship skills
i41	Self-control	Self-control	Self-management
i42	Engagement and relationship skills	Cooperation	Relationship skills
i43	Engagement and relationship skills	Engagement	Relationship skills
i44	Engagement and relationship skills	Responsibility	Responsible decision making
i45	Engagement and relationship skills	Assertion	Self-awareness
i46	Self-control	Self-control	Self-management

*Note*. The item numbering corresponds to the SSIS Rating Scales, and descriptions reflect the content of the items but are abbreviated to avoid copyright violations. SSIS = Social Skills Improvement System; EGA = exploratory graph analysis; SEL = social and emotional learning.
